# Gleeble-Simulated Ultra-Fast Cooling Unlocks Strength–Ductility Synergy in Fully Martensitic Ti-6Al-4V

**DOI:** 10.3390/ma18194572

**Published:** 2025-10-01

**Authors:** Yaohong Xiao, Hongling Zhou, Pengwei Liu, Lei Chen

**Affiliations:** 1 Department of Mechanical Engineering, University of Michigan, Dearborn, MI 48128, USA; 2 Department of Mechanical Engineering, Mississippi State University, Starkville, MS 39762, USA; 3 College of Materials Science and Engineering, Chongqing University, Chongqing 400044, China; 4 School of Mechanical Engineering, Yanshan University, Qinhuangdao 066004, China

**Keywords:** elasto-viscoplasticity, misorientation angle, full martensite, Gleeble, elongated dimple

## Abstract

In additively manufactured (AM) Ti-6Al-4V, the role of martensitic α′ in governing brittleness versus toughness remains debated, largely because complex thermal histories and other intertwined physical factors complicate interpretation. To isolate and clarify the intrinsic effect of cooling rate, we employed a Gleeble thermal simulator, which enables precisely controllable cooling rates while simultaneously achieving ultra-fast quenching comparable to AM (up to ~7000 °C/s). By varying the cooling rate only, three distinct microstructures were obtained: α/β, α_m_/α′, and fully α′. Compression tests revealed that the ultra-fast-cooled fully martensitic Ti-6Al-4V attained both higher strength and larger fracture strain, with densely distributed elongated dimples indicative of ductile failure. Three-dimensional microstructures reconstructed from microscopy, analyzed using an EVP-FFT crystal plasticity model, demonstrated that refined α′ laths and abundant high-angle boundaries promote more homogeneous strain partitioning and reduce stress triaxiality, thereby delaying fracture. These results provide potential evidence that extreme-rate martensitic transformation can overcome the conventional strength–ductility trade-off in Ti-6Al-4V, offering a new paradigm for processing titanium alloys and AM components with superior performance.

## 1. Introduction

Ti-6Al-4V (T64) is one of the most widely used titanium alloys in aerospace and biomedical applications due to its excellent fatigue resistance, high strength-to-weight ratio, toughness, corrosion resistance, and biocompatibility. Traditionally, α/β Ti-6Al-4V microstructures have been manufactured by melting, casting, and secondary processes such as hot isostatic pressing (HIP). However, with the rapid advancement of additive manufacturing (AM) techniques such as selective laser melting (SLM) and direct laser deposition (DLD), these conventional methods are increasingly being complemented or replaced. AM offers net-shape fabrication with high design freedom, low material waste, and the ability to produce complex geometries, making it particularly attractive for on-demand and customized Ti-6Al-4V components [1].

In AM, the tiny melt pool generated during layer-wise printing inherently introduces very high cooling rates (on the order of ~10^5^ K s^−1^), which frequently result in the formation of martensitic α′ microstructures in as-built Ti64 [2]. Traditionally, α′ has been regarded as intrinsically brittle and thus responsible for reduced ductility, leading to extensive efforts to suppress or decompose it through in situ α′ → α + β transformation by process parameter tuning [3], substrate preheating [4], or post-build heat treatments [5]. However, the blanket assumption that α′ is necessarily detrimental to ductility merits careful re-examination. A number of studies [6,7,8,9] have reported that Ti64 containing mixed α + α′—and even fully α′—can achieve ductility comparable to conventional α/β Ti64. De Formanoir et al. [10] showed that dual-phase α/α′ Ti64 produced by sub-transus heat treatment of AM-built material exhibited elongations up to ~22%. Zafari et al. [11] tuned SLM parameters to obtain fully α′ Ti64 with yield strength and tensile elongation comparable to the best commercial wrought α/β Ti64. Zou et al. [12] further indicated that refining prior-β grains can simultaneously enhance strength and ductility. Despite these promising results, AM is not ideal for isolating the intrinsic role of cooling rate in martensitic Ti64 because (1) multiple sources of uncertainty hinder precise control of the product uniformity [13]; (2) the complex multi-physical phenomena in AM (including rapid thermal cycles and melt-pool dynamics [2,14,15] confound the interpretation, in particular, the mechanistic interpretation of how ultra-fast cooling rate alone governs martensitic transformation and mechanical response.

To bypass the above limitations and decouple the cooling rate from other AM physics, we employ a Gleeble 3500D thermal simulator (Dynamic Systems Inc., Poestenkill, NY, USA) [16,17,18]. Unlike AM, the Gleeble platform offers precisely controllable cooling rates in a uniform, well-instrumented environment while still achieving ultra-fast cooling rates comparable to AM (up to ~7000 °C s^−1^). Prior work has leveraged Gleeble-mimicked AM thermal cycles to reproduce microstructures otherwise accessible in AM, thereby validating its fidelity to AM-like kinetics [2]. In the present study, Gleeble enables us to produce uniform Ti64 microstructures—lamellar α/β (slow cooling), mixed α_m_/α′ (intermediate cooling), and fully α′ (ultra-fast cooling)—solely by varying the cooling rate, thus isolating its effect on microstructure and properties. Despite this capability, a systematic verification that pure α′ Ti64 can exhibit excellent ductility—benchmarked directly against α/β and α_m_/α′ counterparts obtained under isolated cooling-rate control—has remained lacking.

Concurrently, the development of fast Fourier transform-based elasto-viscoplastic (EVP-FFT) crystal plasticity modeling provides an effective and computationally efficient framework to interrogate local micromechanics in plastically deforming heterogeneous polycrystals using 3D reconstructed microstructures [19,20]. For Ti-alloy systems, Ozturk et al. [21] built synthetic 3D microstructures from experimental images to assess structure–property relations in α/β Ti-6Al-4V, while Liu et al. [22] integrated FEM, phase-field modeling, and EVP-FFT to study AM Ti-6Al-4V with varying lamellar α/β architectures. These advances enable quantitative linkage from microstructure morphology (e.g., phase fractions) to macroscopic yield behavior, strain partitioning, and damage precursors across α/β, α_m_/α′, and fully α′ Ti64.

Herein, we first aim to verify whether an extremely high cooling rate (~7000 °C s^−1^) can indeed enable a fully martensitic Ti64 to achieve a superior combination of strength and ductility. To this end, we systematically compare the mechanical response of ultra-fast-cooled α′ microstructures with that of α/β and α_m_/α′ states obtained at lower cooling rates. After establishing this performance advantage, we further investigate the underlying micromechanical mechanisms. Specifically, phases and features are characterized by optical and scanning electron microscopy and a piezo-tribo scanner, enabling reconstruction of three distinct 3D digital microstructures as inputs to an EVP-FFT crystal plasticity model. By calibrating the model against compression experiments, we quantify the effects of morphology on yield behavior, strain partitioning, and stress triaxiality. We demonstrate that a high density of high-angle grain/phase boundaries and refined α′ laths facilitates more homogeneous strain distribution and suppresses localized damage, thereby explaining the enhanced ductility of martensitic Ti64 under extreme cooling. These insights establish a cooling-rate-driven paradigm for engineering martensitic Ti-alloys—and AM components—with simultaneously high strength and ductility.

## 2. Materials and Experimental Procedures

**Figure 1:**
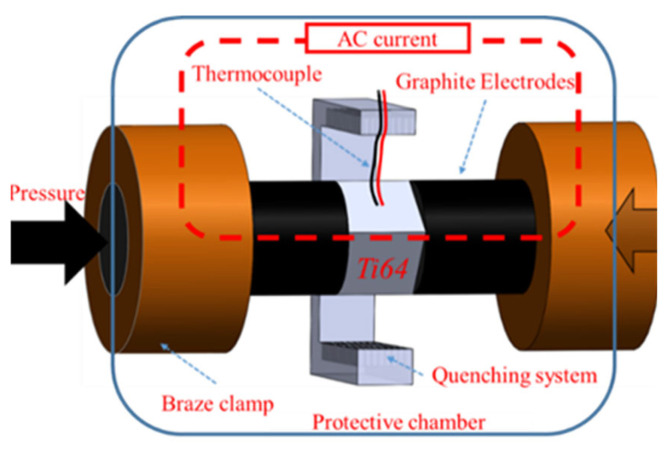
Schematic diagram of the Gleeble 3500D setup with assembled Ti64 sample [2]. The left-pointing arrow on the right side denotes the applied pressure direction during Gleeble thermal simulation.

Commercial Ti-6Al-4V (Ti64) plates (Sigma-Aldrich Inc., St. Louis, MO, USA) were machined into 9.0 × 9.0 × 9.0 mm^3^ cubes using a precision diamond saw. Each cube was sandwiched between graphite electrodes and subjected to a constant force of approximately 5.0 kPa to ensure full contact in the Gleeble 3500D thermal simulator, as illustrated in Figure 1, similar to our previous study [2]. Throughout the heating and cooling processes, the chamber was filled with high-purity argon to suppress oxidation. The samples were rapidly heated to 1100 °C by direct electrical resistance, held for 10 min to guarantee complete transformation to the β phase, and subsequently cooled under three distinct conditions: (i) furnace/grip cooling (Case 1), (ii) air quenching (Case 2), and (iii) fast water-flow quenching (Case 3). The corresponding in situ time–temperature curves can be obtained to quantify the cooling rate.

After cooling, each sample was sectioned to expose a fresh cross-section, which was ground using a Struers Tegrapol-11 polisher (Cleveland, OH, USA) followed by fine polishing with a Buehler VibroMet-2 (Lake Bluff, IL, USA). Phase analysis was performed by X-ray diffraction (XRD). Samples were subsequently etched in Kroll’s reagent (2 mL HF, 4 mL HNO_3_, 50 mL H_2_O; 25 s) and ultrasonically cleaned before microstructural characterization. Optical microscopy (OM) and scanning electron microscopy (SEM) were employed to reveal microstructural morphology. After further vibration polishing, electron backscatter diffraction (EBSD) was performed using a ZEISS SUPRA-40 field emission SEM (Carl Zeiss AG, Oberkochen, Germany) equipped with an EDAX Hikari EBSD camera (AMETEK, Berwyn, PA, USA). Data were analyzed with TSL OIM™ software, version 6.2, with a representative ~1000 × 1000 μm^2^ area scanned at a 2 μm step size to obtain grain misorientation statistics. In addition, topographic scanning was performed with the piezo-tribo scanner in a Hysitron Triboindenter (scanning area: 40 × 40 μm^2^) (Bruker Hysitron, Billerica, MA, USA), enabling quantitative analysis of phase morphology and lath dimensions. Phase size and grain morphology were further quantified using ImageJ 1.52 software.

Mechanical properties were evaluated by compression testing on an Instron 5882 machine (Instron, Norwood, MA, USA) equipped with a 25 mm extensometer. Tests were conducted at room temperature with a constant strain rate of 0.001 s^−1^. For each condition, four replicate samples were tested to ensure reproducibility, and average values are reported. It is noted that the small cubic geometry was intentionally selected to facilitate the achievement of extremely high cooling rates and uniform microstructures, making compression testing more reliable than tensile testing under these conditions.

## 3. Model Descriptions

### 3.1. FFT Based Elastic-Viscoplastic Self-Consistent Model

The detailed formulation of the fast Fourier transform (FFT)-based elasto-viscoplastic (EVP-FFT) approach has been reported elsewhere [19]. Here, we briefly summarize the key theoretical framework adopted in this study. The total strain field **ε**(**x**) can be written as follows:

(1) $$\varepsilon \left(x\right)=C^{-1}\left(x\right):\sigma \left(x\right)+\varepsilon^{p,t}\left(x\right)+{\dot{\varepsilon }}^{p}\left(x\right)∆t$$

where **C**(**x**) is the elastic stiffness tensor, **ɛ^p^**(**x**) is the plastic strain tensor. The plastic strain-rate ${\dot{\varepsilon }}^{p}\left(x\right)$ is a constitutive relation with stress **σ**(**x**) at a single crystal material point **x** by a sum over the *N* active slip systems, of the form:

(2) $${\dot{\varepsilon }}^{p}\left(x\right)=\sum_{s=1}^{N}m^{s}\left(x\right){\dot{\gamma }}^{s}\left(x\right)={\dot{\gamma }}_{0}{\sum_{s=1}^{N}m^{s}\left(x\right)\left(\frac{\left|m^{s}\left(x\right):\sigma \left(x\right)\right|}{\tau_{0}^{s}\left(x\right)}\right)}^{n}\mathrm{sgn}\left(m^{s}\left(x\right):\sigma \left(x\right)\right)$$

where ${\dot{\gamma }}^{s}\left(x\right)$, ${\dot{\gamma }}_{0}$ and $m^{s}\left(x\right)$ are, respectively, the shear rate, normalization factor, and Schmid tensor of slip systems, $\tau_{0}^{s}\left(x\right)$ is the critical resolved shear stress (CRSS), *n* is a stress exponent, namely, the inverse of the rate-sensitivity exponent.

The stress **σ**(**x**) can be written as:

(3) $$\sigma_{ij}\left(x\right)=\varphi_{ij}\left(x\right)+C_{ijkl}^{0}\left(x\right)u_{k,l}\left(x\right)=\varphi_{ij}\left(x\right)+C_{ijkl}^{0}\left(x\right)\varepsilon_{kl}\left(x\right)$$

where *u_k_*_,*l*_(**x**) represents the displacement gradient tensor and *ε_kl_*(**x**) = (*u_k_*_,*l*_(**x**) + *u_l_*_,*k*_(**x**))/2, $\varphi_{ij}\left(x\right)$ is the polarization field. Combining Equation (3) with the equilibrium equation (*σ_ij_*_,*j*_(**x**) = 0):

(4) $$C_{ijkl}^{0}\left(x\right)u_{k,lj}\left(x\right)+\varphi_{ij,j}\left(x\right)=0$$

The differential Equation (4) for a unit cell with periodic boundary conditions and an initial strain can be solved using the Green function method. As the periodic Green function *G_km_*(**x**) is related to the displacement field *u_k_*(**x**), the solution of *u_k_*(**x**) is obtained by the convolution:

(5) $$u_{k,l}\left(x\right)=\int_{R^{3}}G_{ki,jl}\left(x-x^{\prime }\right)\varphi_{ij}\left(x^{\prime }\right)dx^{\prime }$$

Further transferring Equation (5) into Fourier space by the convolution theorem, the compatible strain field *ε_ij_*(**x**) deriving from the solution of Equation (4) is given by:

(6) $$\varepsilon_{ij}\left(x\right)=E_{ij}+{\mathrm{FT}}^{-1}\left(sym\left({\hat{\Gamma }}_{ijkl}^{0}\left(\xi \right)\right){\hat{\varphi }}_{kl}\left(\xi \right)\right)$$

where the symbol of “^” and “FT^−1^” indicate the Fourier transform and its inverse transformation, respectively, **ξ** is a frequency point in Fourier space ${\hat{\Gamma }}_{ijkl}^{0}\left(\xi \right)=-\xi_{j}\xi_{l}{\hat{G}}_{ik}\left(\xi \right)$, the Green operator in Fourier space is a function of the reference stiffness tensor and frequency, in which ${\hat{G}}_{ik}\left(\xi \right)={\left[C_{kjil}^{0}\xi_{l}\xi_{j}\right]}^{-1}$.

Equation (6) is a fix-point equation for the strain field that allows solving the constitutive and governing equations iteratively. In practice, for better convergence of our micromechanical model, we use a modified version of the above algorithm, based on the augmented Lagrangian formulation of Michel et al. [23], adapted to the EVP polycrystals (see [19] for details).

### 3.2. Voce-Type Hardening Law

Considering the strain hardening, the extended Voce Law [24,25] is used to describe the relationship between threshold stress *τ^s^* and total accumulated shear strain *Γ*, given by:

(7) $$\tau^{s}\left(\Gamma \right)=\tau_{0}^{s}+\left(\tau_{1}^{s}+\theta_{1}^{s}\Gamma \right)\left(1-\exp\left(-\Gamma \left|\frac{\theta_{0}^{s}}{\tau_{1}^{s}}\right|\right)\right)$$

where *θ*_0_ and *θ*_1_ are the initial and asymptotic hardening rates, respectively, (*τ*_0_ + *τ*_1_) refers to the back-extrapolated stress. The elastic constants of β, α, and α′ phases and hardening parameters used for the Voce model are further calibrated in Section 4.2.2, correlating with compression test results.

## 4. Results

### 4.1. Experimental Results

#### 4.1.1. Phase and Microstructure Analysis

**Figure 2:**
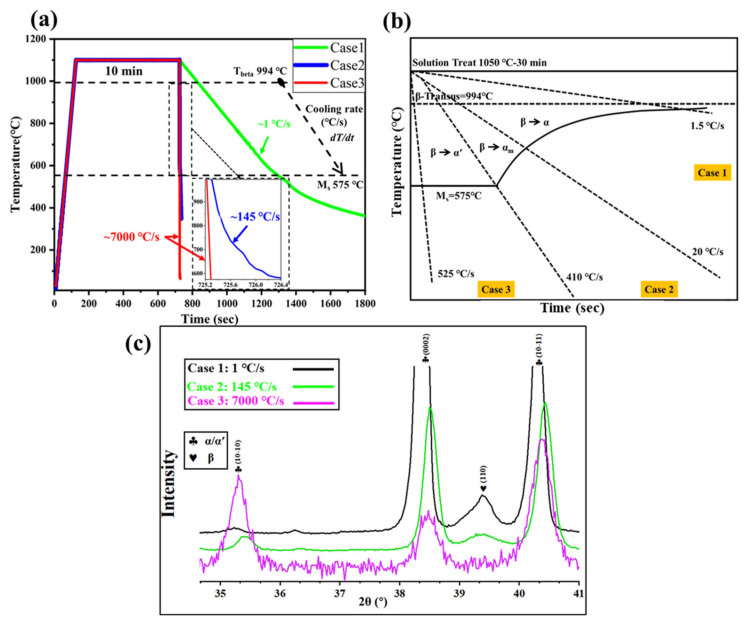
(**a**) In situ time–temperature curves and average cooling rates between β-transus and martensite start temperature; (**b**) Schematic cooling diagram of Ti-6Al-4V [26]; (**c**) XRD patterns of Ti64 under different cooling conditions.

**Figure 3:**
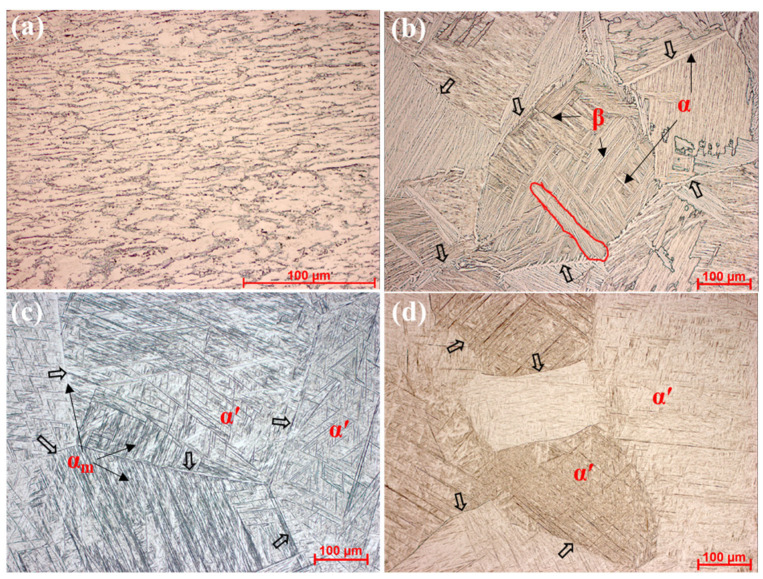
Optical microscopy (OM) images of etched samples with different cooling rates: (**a**) as-received sample, (**b**) Case 1: 1 °C/s, (**c**) Case 2: 145 °C/s, and (**d**) Case 3: 7000 °C/s. Hollow arrows indicate prior-β boundaries, and red outlines highlight colony structures.

The real-time heating and cooling histories under the three Gleeble-controlled conditions are presented in Figure 2a, and the corresponding cooling-rate windows between the β-transus and the martensite start temperature are summarized in Figure 2b [26]. Before cooling, all phases in the as-received samples were transformed to β due to 1100 °C > T_β_ = 994 °C, and the 10 min holding at 1100 °C ensured a complete α + β → β transformation. As illustrated in Figure 2a, subsequent cooling produced three distinct conditions with approximate average cooling rates of 1 °C/s, 145 °C/s, and 7000 °C/s, corresponding to α/β (Case 1), α_m_/α′ (Case 2), and fully α′ (Case 3) microstructures, respectively, as predicted by the Ti-6Al-4V cooling diagram (Figure 2b). These predictions are verified by XRD (Figure 2c): Case 1 retains both α and β peaks, consistent with a lamellar α/β structure; Case 2 shows weakened β peaks with broadened and shifted α peaks, indicative of coexisting massive α and martensitic α′; Case 3 exhibits only α′, confirming a fully martensitic transformation at the highest cooling rate. Although α and α′ share similar lattice structures, their morphological differences are clarified in etched OM micrographs (Figure 3).

Figure 3 further illustrates the morphological differences among cases. The as-received sample (Figure 3a) shows a typical rolled structure consisting of ellipsoidal α plates delineated by thin β films. After Gleeble treatment, prior-β grains coarsened to ~400 μm and changed shape from ellipsoidal to polygonal due to the 10 min holding at 1100 °C. Across all cases, the prior-β grain size remained ~400 μm, reflecting the identical heating schedule. Case 1 (Figure 3b) presents classical lamellar α/β colonies nucleated preferentially at prior-β boundaries (indicated by hollow arrows). In Case 2 (Figure 3c), massive α plates distribute mainly along prior-β boundaries and within some grains, while α′ laths fill the remaining regions, forming an α_m_/α′ dual microstructure. Case 3 (Figure 3d) shows sharp prior-β interfaces without boundary α films, characteristic of a fully martensitic structure.

In detail, Case 1 exhibits a typical lamellar α/β structure composed of numerous α colonies. As indicated by the hollow arrows in Figure 3b, prior-β grain boundaries serve as preferential nucleation sites for α plates, which subsequently grow inward to form colony structures (outlined by red lines) [27]. The XRD pattern of Case 1 (Figure 2c) also shows pronounced β peaks, confirming a significant β fraction. By contrast, Case 3 (Figure 3d) reveals no α boundary layers; instead, sharp prior-β interfaces are preserved (black arrows), consistent with a fully martensitic transformation and with the absence of β reflections in Figure 2c, Case 3 [28]. Case 2 (Figure 3c) represents an intermediate state where massive transformation and martensitic transformation occur simultaneously, leading to a mixed α_m_/α′ microstructure. The α_m_ plates are mainly distributed along prior-β boundaries and partially within grains [26], while α′ laths occupy the same β grains. The residual β fraction in Case 2 is extremely small, reflected by weak β peaks in Figure 2c; this β is typically retained within bulk α, i.e., the α_m_ phase [2]. Moreover, the presence of α′ broadens and slightly shifts the α/α′ reflections, further confirming that both Case 2 and Case 3 contain martensitic α′. To characterize these phase features in detail, topography and SEM imaging were conducted to quantify morphology—including thickness, length, and volume fraction—as summarized in Table 1.

**Figure 4:**
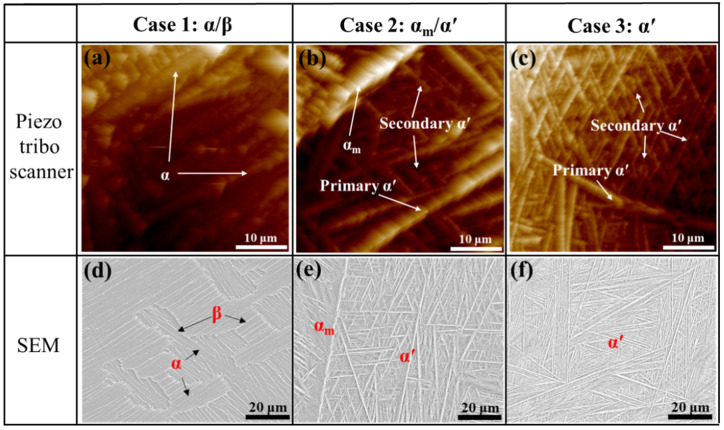
Surface topography images (**a**–**c**) and SEM images (**d**–**f**) of etched samples: (**a**,**d**) Case 1, (**b**,**e**) Case 2, and (**c**,**f**) Case 3.

To quantify phase features, surface topography, and SEM images (Figure 4) were analyzed. Two α′ morphologies were distinguished: primary α′, coarse parallel-sided needles adjacent to prior-β boundaries, and secondary α′, ultrafine needles distributed within grains. A remarkable primary α′ was captured by piezo-tribo scanning at the prior-β boundary in Case 3 (Figure 4c), confirming its significantly larger thickness and length compared to secondary α′. Comparing Case 2 (Figure 4b) and Case 3 (Figure 4c), both primary and secondary α′ became shorter and finer as the cooling rate increased. With the ImageJ 1.52 software, quantitative thickness, length, and volume fraction of phases are summarized in Table 1, which also served as input data for reconstructing digital microstructures for EVP-FFT modeling (see Section 4.2.1).

#### 4.1.2. Compression Testing and Work Hardening Behaviors

**Figure 5:**
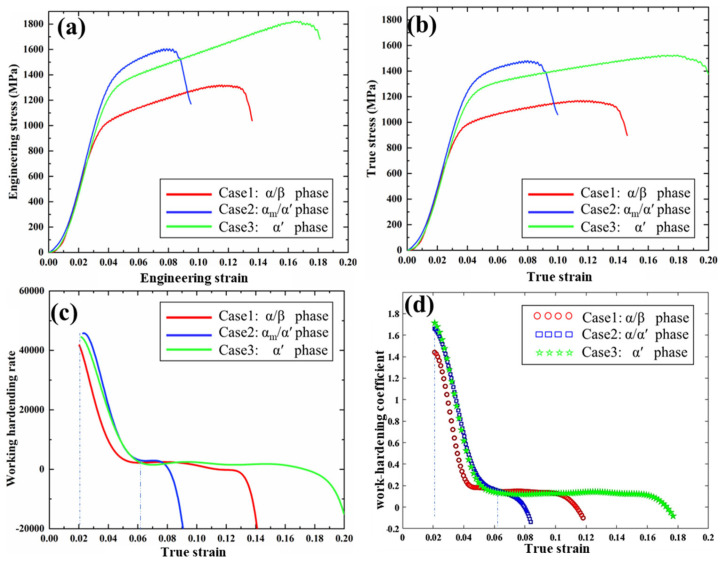
Compression tests of Ti-6Al-4V under different cooling conditions: (**a**) engineering stress–strain curves, (**b**) true stress-strain curves, (**c**) work-hardening rate (θ) as a function of true strain, and (**d**) work-hardening coefficient (*n*) as a function of true strain.

The engineering compressive stress–strain curves up to fracture are shown in Figure 5a. Two characteristic points are of primary interest: the yield point and the ultimate compressive point. Because Ti-6Al-4V does not exhibit an obvious yield drop under compression, the 0.2% offset method was applied to define the compressive yield strain (ε_cy0.2_) and yield stress (σ_cy0.2_). The maximum stress sustained at fracture is reported as the ultimate compressive strength (σ_cu_), corresponding to the ultimate strain (ε_cu_).

As summarized in Table 2, Case 1 (α/β, 1 °C/s) starts to yield at ε_cy0.2_ ≈ 0.029 with σ_cy0.2_ ≈ 790 MPa, close to the value reported in [29]. At ε_cu_ ≈ 0.12, shear fracture initiates along a 45° plane with σ_cu_ ≈ 1161 MPa. In Case 2 (α_m_/α′, 145 °C/s), both ε_cy0.2_ and σ_cy0.2_ increase to ~0.039 and ~1215 MPa, respectively. However, fracture occurs earlier, at ε_cu_ ≈ 0.08, although the corresponding σ_cu_ rises to ~1478 MPa, higher than that in Case 1. With further increase in the cooling rate to 7000 °C/s (Case 3, fully α′), the highest ε_cu_ ≈ 0.17 and σ_cu_ ≈ 1519 MPa are obtained simultaneously. The yield point of Case 3 is ε_cy0.2_ ≈ 0.037 with σ_cy0.2_ ≈ 1074 MPa. These results indicate that full martensitic transformation induced by extremely high cooling rate enables Ti-6Al-4V to achieve both excellent ductility and high compressive strength.

The true (logarithmic) stress–strain curves (Figure 5b) were further used for calibration of the EVP-FFT model [30]. The yield behaviors are more clearly reflected in Figure 5c,d, which plot the work-hardening rate (θ=∂σ/∂ε) and work-hardening coefficient (n=∂lnσ/∂lnε), respectively. According to Hollomon’s equation [10,31], both curves show a similar trend as reported in [32]: a sharp decrease occurs before ε ≈ 0.06, corresponding to the transition from elastic to plastic regimes [33], followed by a relatively stable stage until fracture. During the initial drop, θ and *n* values for Case 2 and Case 3 remain higher than those of Case 1, consistent with their higher yield strengths as listed in Table 2.

To further understand the micromechanical origins of these trends, the EVP-FFT model was applied to probe strain partitioning and stress triaxiality in the α/β, α_m_/α′, and fully α′ microstructures (Section 4.2).

#### 4.1.3. Fractographic Analyses

**Figure 6:**
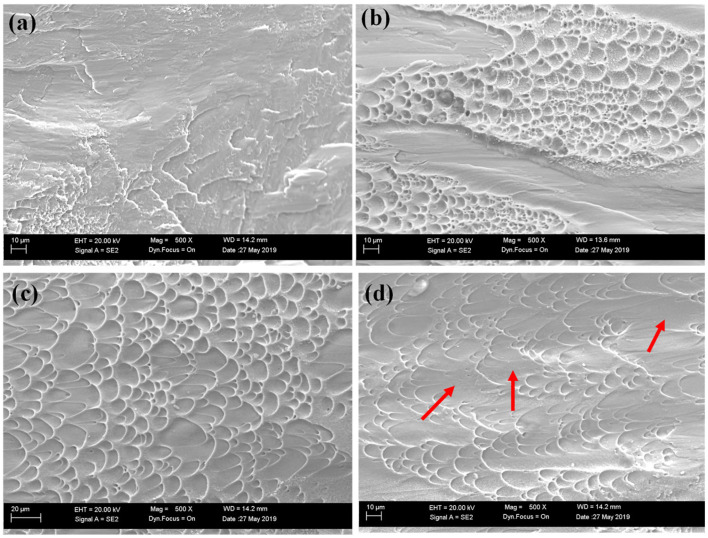
Fractographs of samples compressed under different cooling conditions: (**a**) Case 1 (1 °C/s, α/β), (**b**) Case 2 (145 °C/s, α_m_/α′), and (**c**,**d**) Case 3 (7000 °C/s, fully α′).

To examine fracture features under different cooling conditions, SEM fractographs were captured, as shown in Figure 6. A clear distinction is observed between Case 1 and the other two cases. In Case 1 (α/β, 1 °C/s), fracture surfaces lack obvious dimpled regions, unlike Cases 2 and 3. However, Case 1 does not exhibit a typical brittle fracture, as no transgranular or intergranular cleavage is visible. Instead, failure likely initiates by tearing along weak β regions under compression, consistent with the inherently low strength but high ductility of the β phase. In contrast, dimpled zones are clearly observed in Cases 2 and 3. Moreover, the dimples in Case 3 are larger and more elongated than those in Case 2, which indicates stronger plastic accommodation in the fully martensitic microstructure. Such elongated dimples in Ti64 have also been reported under dynamic compression at a strain rate of ~3900 s^−1^ using a split Hopkinson pressure bar, where they were associated with severe plastic deformation inside adiabatic shear bands [34].

It is well known that spherical dimples correspond to micro-voids that nucleate cracks [35]. In Figure 6b,c, numerous micro-voids are visible at the intersections of dimples. In Case 3 (Figure 6d), elongated dimples coalesce and evolve into smeared surfaces (indicated by red arrows), suggesting that the fully martensitic α′ structure can sustain severe plastic deformation even after crack initiation. By contrast, in Case 2, the α_m_/α′ structure fails to accommodate further deformation once cracking begins, consistent with compression testing results showing that Case 3 exhibits much higher compressive strain to failure than Case 2.

### 4.2. Modeling the Crystal Plasticity of Different Phase Compositions

#### 4.2.1. Reconstructed 3D Grain Structures

**Figure 7:**
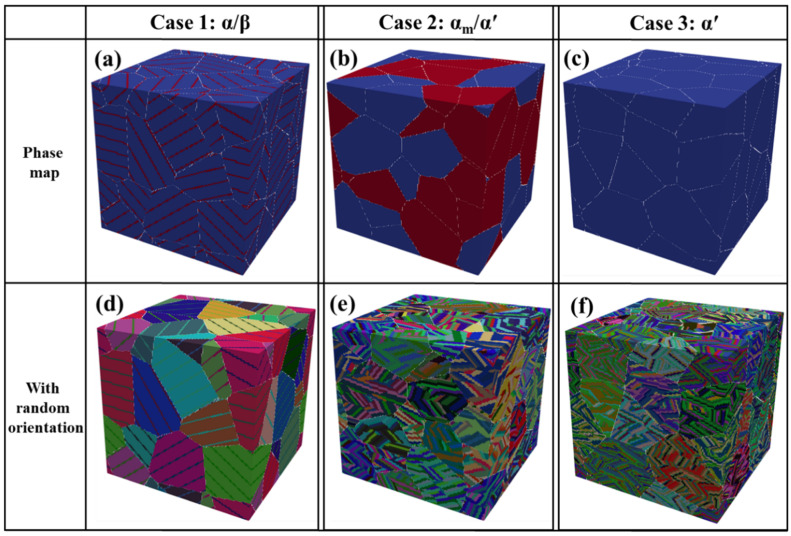
Reconstructed 3D microstructures (64^3^ domain size) with different phase compositions. (**a**–**c**) phase maps, and (**d**–**f**) random orientation maps: (**a**,**d**) Case 1 (1 °C/s, α/β), (**b**,**e**) Case 2 (145 °C/s, α_m_/α′), and (**c**,**f**) Case 3 (7000 °C/s, primary/secondary α′).

Figure 7 was generated using the MTEX toolbox in Matlab version 9.6 [22,36] based on the morphological information listed in Table 1. The phase distributions of reconstructed 3D microstructures for the three cooling conditions are shown in Figure 7a–c. Specifically, Figure 7a represents the lamellar α/β microstructure, where α plates nucleate preferentially along prior-β boundaries (white lines) and extend into the β matrix. The inner α (blue blocks in Figure 7a) and β (red blocks) form colonies of α/β lamellae following the Burgers orientation relation (BOR) [22]. For simplicity, the competitive colony growth inside individual grains was not considered in the Case 1 model.

Figure 7b shows the α_m_/α′ microstructure (Case 2). Here, α_m_ plates (blue blocks) are distributed mainly along prior-β boundaries (white lines) and occupy part of the grains, while the remaining regions are filled with martensitic α′ (red blocks). Figure 7c corresponds to the fully martensitic microstructure (Case 3). In this case, a small fraction of long primary α′ (white lines) is assigned at prior-β boundaries, whereas the interiors of the grains are occupied by dense secondary α′ laths (blue blocks).

It should be noted that crystallographic textures are not emphasized in the present reconstruction; phases are therefore assigned random orientations, as illustrated in Figure 7d–f. This approach approximates the chaotic distribution of needle-like martensite observed experimentally in Figure 4c,d. To mimic this effect, prior-β grains were subdivided into smaller zones that were filled with randomly oriented secondary α′ needles.

**Figure 8:**
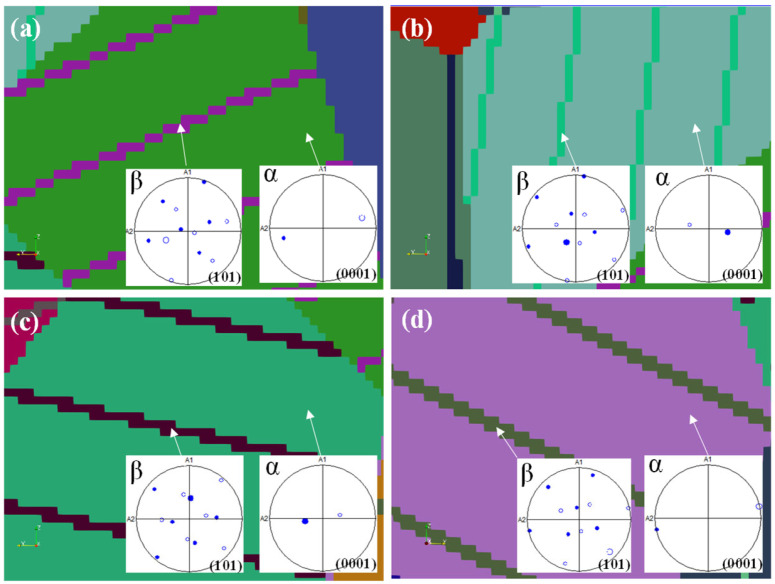
Four randomly selected examples (**a**–**d**) regarding pole-figure verification of the BOR relationship in reconstructed α/β lamellae: (101)β plane aligned with (0001)α plane after simulated rotations.

The crystallographic relationship between α and β laths is maintained through the Burgers orientation relation (BOR), namely, (0001)_α_//{101}_β_ and <$11\bar{2}0$>_α_/<111>_β_ [37]. This was verified by the symmetric rotation tool in TSL OIM Analysis. Figure 8 shows a selected α/β lamellar region in the reconstructed 3D model and the corresponding pole figures obtained after applying the appropriate rotation angles. The result confirms that the reconstructed lamellae preserve the BOR relationship, specifically (0001)_α_//{101}_β_.

#### 4.2.2. Calibration of Simulation Compression Curves

**Figure 9:**
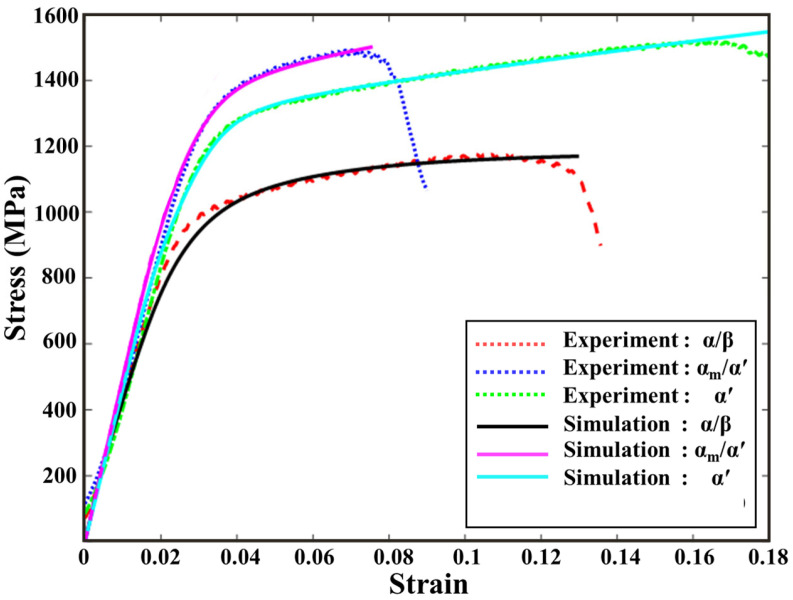
Experimental and EVP-FFT simulated compression stress–strain curves for samples with different phase/microstructure combinations.

Due to the lack of phase-specific elastic constants and hardening parameters measured in compression, the literature values are primarily obtained from tensile tests [21,38] were used as initial priors and then calibrated against the present compression experiments (Figure 5b) for each microstructure. A working hypothesis adopted in the calibration is *τ*_0_ of β ≪ α′ < α as listed in Table 3 and Table 4. This ordering is motivated by repeated nanoindentation evidence showing that single-phase α′ is softer than single-phase α [10]. Although this may appear to contradict the popular notion that “α′ is harder than α.” The possible reason is that the indentations of general hardness tests like Vikers hardness tests are micro-scale and likely incorporate much α′ phase. Therefore, other factors like dislocations and phase boundaries may contribute to an elevated hardness. Hence, for Case 3, a small *τ*_0_ is reasonably used for calibration. Otherwise, the strain-stress curve of pure martensite is more likely to yield at a higher strain, shown in Figure 9 simulation-α′ (2). It should be noted that the α_m_ is regarded as the bulk α [2], where the very small amount of β distributed in the bulk α is ignored therein, and the parameters of the bulk α phase are calculated by considering the thickness [22].

Figure 9 compares the computational and experimental stress–strain curves for the three cases. The simulations reproduce both the elastic-to-plastic transition and the relative work-hardening trends across α/β (Case 1), α_m_/α′ (Case 2), and fully α′ (Case 3). Fracture is not modeled explicitly here due to the absence of a failure criterion; damage precursors and stress-triaxiality analyses are deferred to Section 5.

#### 4.2.3. Overall Compressive Strain Field

Because of the anisotropy of the HCP structure, three <$11\bar{2}0$> (0001) basal, three <$11\bar{2}0$>{$10\bar{1}0$} prismatic, six <$11\bar{2}0$>{$10\bar{1}1$} **<α>** pyramidal slip systems for the HCP phase (α′/α phases) are considered in the crystal plasticity computation. It should be noted that six <$11\bar{2}3$>{$10\bar{1}1$} **<α+c>** pyramidal slip systems are excluded because they are unlikely to be activated [39]. At the same time, twelve <111> {110} slip systems for the BCC phase (β phase) are integrated into this model. Twinning is not considered, as deformation twinning in Ti-6Al-4V is rarely observed at ambient temperature and low strain rates [37,40,41].

**Figure 10:**
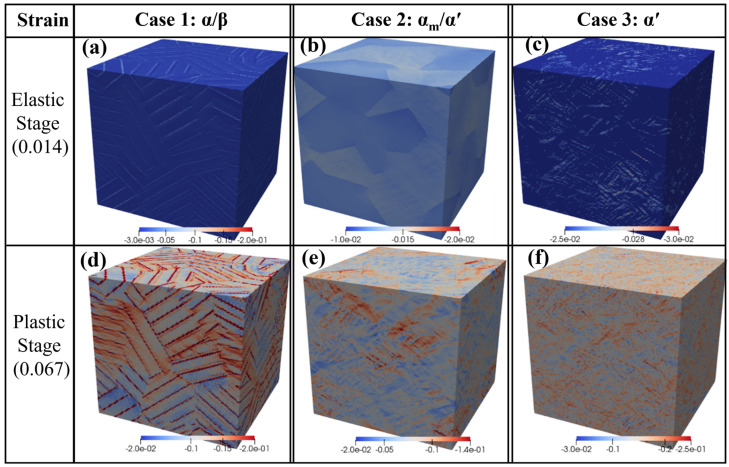
Simulated 3D maps of elastic and plastic strain at true strains of 0.014 (**a**–**c**) and 0.067 (**d**–**f**), respectively: (**a**,**d**) Case 1 (α/β), (**b**,**e**) Case 2 (α_m_/α′), and (**c**,**f**) Case 3 (primary/secondary α′). Negative values correspond to compressive strain.

Figure 10 shows the simulated 3D elastic and plastic responses after the compression strain of 0.014 (elastic stage) and 0.067 (plastic stage), respectively. It can be observed that the distribution of strain concentration varies in both inter- and intra-grains for different cases. Some of the grains after compression exhibit a strong strain concentration, while some exhibit limited strain concentration. In particular, some extremely high strain concentrations take place locally near the grain boundary, indicated by deep color. The inter- and intra-phases also show a similar trend of strain concentration. However, the strain/stress concentration varies from grain to grain and from phase to phase. Investigation of the plastic behavior of different phases may provide significant insights as to how grain and phase interactions affect the micromechanics of slip systems, phase species, and morphologies. Because grain and phase boundaries are often the locations where plastic deformation is most pronounced. Moreover, since the reconstructed microstructure does not fully capture the precise morphology and the model does not explicitly include dislocations, the analysis does not account for the role of dislocations in plastic behavior. Instead, the stress and strain concentration coupled with the aforementioned slip systems are used to describe the role of different phases and morphologies on micro-mechanical responses.

## 5. Discussion

In this work, Ti64 quenched from above the β-transus at an ultra-high cooling rate of ~7000 °C/s exhibits a concurrent improvement in ductility and strength. This observation challenges the common perception of a strength–ductility trade-off, although several studies have reported excellent performance for fully martensitic Ti64 [11,12] without a systematic mechanistic analysis. Here, we discuss the phase-dependent micromechanics underlying the different behaviors among α/β (Case 1), α_m_/α′ (Case 2), and fully α′ (Case 3), with emphasis on why the fully martensitic structure obtained under AM-level cooling rate attains enhanced ductility relative to α/β and α_m_/α′. These insights suggest a processing paradigm for Ti alloys that achieves high strength and high ductility simultaneously. Methodologically, instead of digital image correlation (DIC), we leverage the EVP-FFT model built on 3D reconstructed microstructures to provide quantitative descriptions of strain partitioning and stress triaxiality—two metrics that capture mechanical contrast among phases and morphologies and link directly to ductile fracture propensity.

### 5.1. A Quantitative Understanding of the Yield Behaviors for Different Cases

From Table 1, the characteristic phase size in Case 2 is ~7× larger than in Case 1, which would ordinarily reduce yield strength according to Hall–Petch scaling [42]. Yet the measured yield stress increases by ~1.35× (Figure 5c,d), which is credited to two possible reasons. One is that the β phase in Case 2 is nearly replaced by α′ and α_m_, both of which are harder-slipping than β due to fewer easy systems and higher CRSS; this is consistent with micro-/nano-scale hardness contrasts reported in [10]. Consequently, the initial yielding is elevated. Another possible reason is that Case 2 contains a far more intricate network of grain/phase boundaries—especially the disordered distribution of α′ laths (Figure 4e)—than the predominantly lamellar α/β in Case 1. Molecular-dynamics simulations have indicated that disordered grain-boundary structures can significantly raise the yield stress by impeding dislocation motion [43].

For Case 3, the yield strength is slightly lower than Case 2 (with marginally lower initial θ and *n* in Figure 5c,d), even though its martensitic laths are finer. This is consistent with the phase effect: single-phase α′ is intrinsically softer than bulk α (α_m_) at the slip-system level [10], so more systems are relatively easier to activate at yielding in Case 3. To quantify these effects, we employed the EVP-FFT model to analyze strain accumulation and partitioning at the yield points of all three cases.

**Figure 11:**
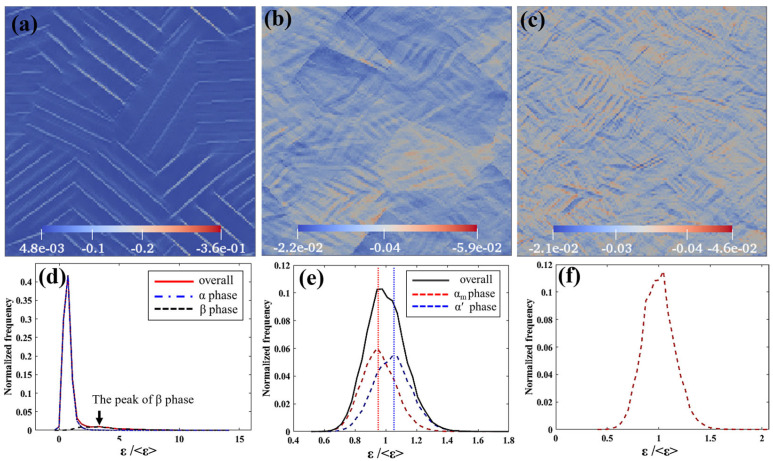
The 2D strain maps sliced from 3D strain models at each yield point for different cases, corresponding to the frequency of strain of each phase normalized by the average strain in the whole domain at the yield point: (**a**,**d**) 0.029 for Case 1; (**b**,**e**) 0.039 for Case 2; (**c**,**f**) 0.037 for Case 3.

Compared with DIC studies [10], the EVP-FFT approach provides field-resolved strain maps at the yield point along the compression axis, enabling direct comparison of local heterogeneity across phases. The color maps in Figure 11a–c show that microstructural diversity leads to heterogeneous strain accumulation in all cases. In Case 1 (α/β), strain partitions strongly between α and β (Figure 11d) [10], concentrating primarily in β because of its higher symmetry and much lower CRSS, with local values up to ~12.4× the macroscopic yield strain—consistent with fracture surfaces torn along β lamellae. In Case 2 (α_m_/α′), localization occurs mainly in α′ (Figure 11e), but the peak is only ~1.5× the yield strain, reflecting smaller CRSS contrasts between α and α′ than between α and β. In Case 3 (fully α′), strain is more uniformly distributed (Figure 11f), and the peak is ~1.2× the yield strain, indicative of reduced partitioning in the refined α′ network.

Overall, the presence of soft β dominates the pronounced strain partitioning and the lowest yield strain in Case 1. Introducing α′ (Case 2) raises the yield level; switching to fully α′ (Case 3) slightly reduces σ_cy0.2_ relative to Case 2 due to α′’s lower CRSS than α, despite finer lath size—consistent with the experimental trends.

### 5.2. Potential Mechanisms for the Marked Ductility Improvement in Fully Martensitic Structures

#### 5.2.1. High-Angle Interfaces and Ductility

**Figure 12:**
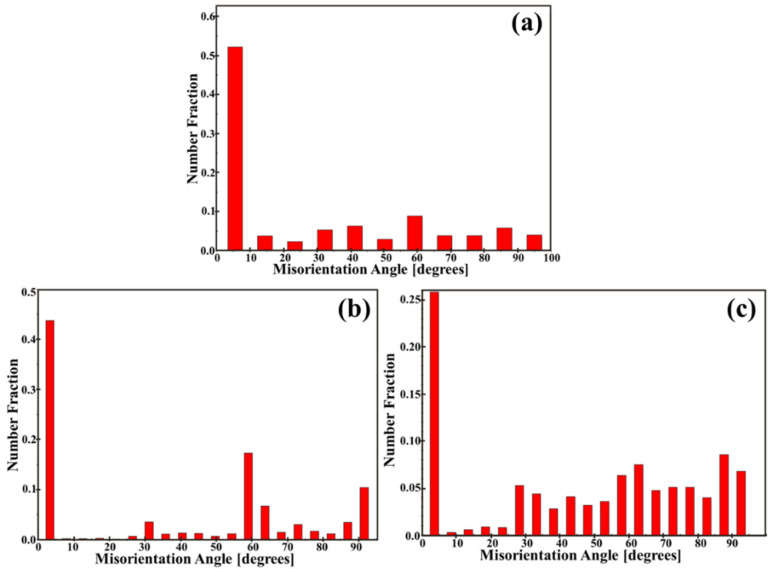
EBSD misorientation distributions: (**a**) Case 1 (α/β), (**b**) Case 2 (α_m_/α′), (**c**) Case 3 (α′).

A high density of high-angle interfaces is a plausible contributor to the superior ductility in Case 3. In this study, low-angle grain boundaries (LAGBs) are defined as boundaries with misorientation angles between 2° and 15°, while high-angle grain boundaries (HAGBs) are defined as those with misorientation angles between 15° and 90° [44]. EBSD misorientation statistics (Figure 12) indicate that increasing the cooling rate raises the fraction of HAGBs: in Case 1, over half of the boundaries are low-angle, whereas in Case 3 ≈70% of boundaries exhibit misorientation even >30%°. Such a population of HAGBs can promote boundary-mediated accommodation—e.g., dislocation absorption and deflection, impeded slip transfer, and crack-tip blunting—thus delaying localization and enhancing macroscopic ductility [5,44].

**Figure 13:**
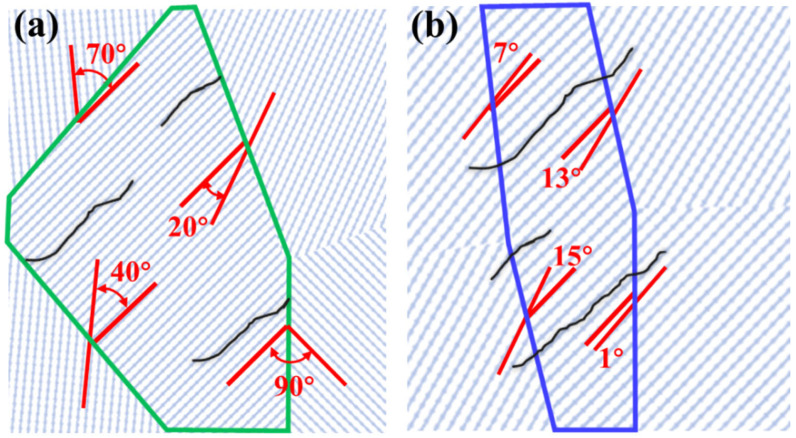
Schematic of boundary effects on crack propagation: (**a**) high-angle misorientation facilitating deflection/blunting; (**b**) low-angle misorientation providing easier crack paths.

Additionally, the ultrafine α′ lath network in Case 3 offers a very high density of internal interfaces. Micro-cracks propagating along one α′ lath are likely to be deflected or arrested upon encountering another lath with a large misorientation (schematized in Figure 13), delaying crack advance until larger global strains are attained. This interpretation is consistent with our EVP-FFT results that show a narrower strain distribution spread in Case 3 (Figure 11c,f) and with the fractography, where Case 3 exhibits longer wedge-like dimples than Case 2 (Figure 6d vs. Figure 6b). Based on our 3D reconstructions, the interfacial area density in Case 3 is estimated to be several-fold higher (on the order of ~6–10×) than in Cases 1–2, which aligns with the observed enhancement in boundary-mediated plastic accommodation [45,46].

#### 5.2.2. Stress Triaxiality Factor

One useful approach to evaluate the stress state during plastic deformation is to track the stress triaxiality factor (TF or η), defined as the ratio of local mean stress (hydrostatic, *σ_m_*) and local equivalent stress (von Mises or effective stress, *σ_vm_*) [47,48]. In tensile tests, it is well documented that increased stress triaxiality reduces ductility. Hancock and Mackenzie [49], for example, demonstrated that the ductility of high-strength steels strongly depends on stress state, characterized by *σ_m_*/*σ_vm_*, which is also consistent with McClintock’s model of ductile failure. Negative values of stress triaxiality indicate compressive stress states [50]. In this study, stress triaxiality was calculated at the fracture point to evaluate potential failure initiation, using EVP-FFT simulations that provide the full stress tensor at every Fourier grid point.

**Figure 14:**
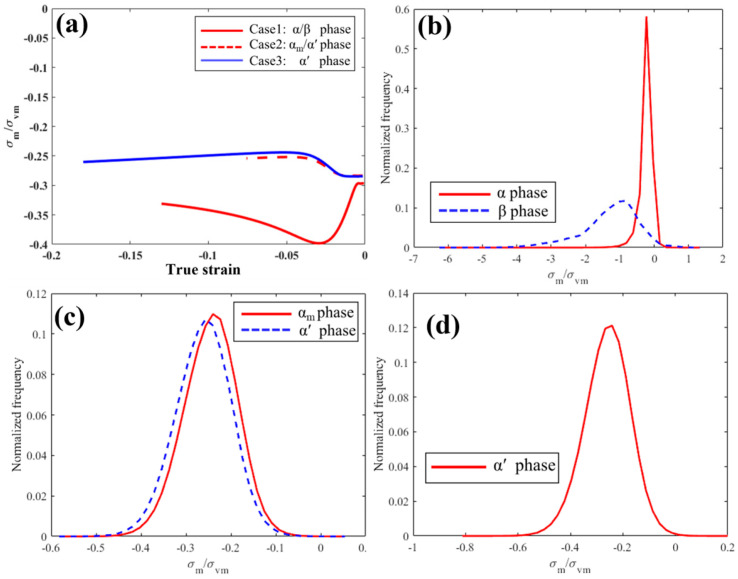
(**a**) The average *σ_m_*/*σ_vm_* variation against true strain and *σ_m_*/*σ_vm_* distributions for different cases: (**b**) Case 1, (**c**) Case 2, and (**d**) Case 3.

The evolution of average TF as a function of true strain up to fracture is shown in Figure 14a. In Case 1, |TF| first increases and then decreases to ~0.35 at fracture. By contrast, Cases 2 and 3 exhibit the opposite trend, with lower |TF| values throughout. At the fracture point, TF is ~0.26 in Cases 2 and 3, indicating that these microstructures experience less severe stress states and therefore resist fracture initiation more effectively than Case 1. The higher |TF| in Case 1 can be attributed to the presence of the soft β phase, which promotes extreme local stress concentrations.

The TF distributions at the fracture point (Figure 14b–d) further illustrate this phase dependence. In Case 1, the β lamellae show the largest |TF|, identifying them as the weakest links, consistent with the fractographic evidence (Figure 6a) where cracks propagate along β. Case 2 shows a similar trend, but with a smaller contrast between α and α′. In contrast, Case 3 exhibits the lowest TF values and the most uniform distribution during compression, correlating with its superior ductility and higher compressive strain to failure.

## 6. Conclusions

In this study, we demonstrated that Ti-6Al-4V quenched from above the β-transus at an extremely high cooling rate (~7000 °C/s) forms a fully martensitic microstructure that simultaneously achieves enhanced strength and ductility compared with α/β and α_m_/α′ microstructures obtained at slower cooling rates. Compression testing confirmed that the fully α′ samples exhibit not only higher ultimate compressive strength and larger strain to failure, but also stronger work-hardening, lower stress-triaxiality magnitude, and fracture surfaces dominated by elongated dimples. By contrast, the α/β and α_m_/α′ conditions display obvious strain partitioning and stress-triaxiality partitioning, as captured quantitatively by EVP-FFT simulations.

Yield behavior was found to depend strongly on phase constitution. The presence of coarse α lamellae and the soft β phase in the α/β microstructure explains its lowest yield strength. With increasing cooling rate, the introduction of α′ significantly raises the yield strength in the α_m_/α′ condition, since β is largely replaced by harder phases. Although the fully martensitic case has the finest lath size, its yield strength is slightly lower than Case 2, because single-phase α′ has a lower CRSS than α. Nevertheless, the fully α′ structure provides the most favorable combination of strength and ductility.

Two microstructural factors appear to be central to the superior ductility of the martensitic Ti64. First, the high fraction of high-angle grain/phase boundaries—about 70% of boundaries with misorientation > 30° in Case 3—promotes boundary sliding and plastic accommodation. Second, the ultrafine α′ lath network produces a very high density of interfaces, which helps deflect and blunt cracks, thereby delaying propagation. These mechanisms, together with possible contributions from dislocation structures formed under rapid cooling, underpin the observed ductility enhancement.

Overall, this work highlights that controlling the cooling rate provides an effective pathway to tailor the phase constitution and interface architecture of titanium alloys. Fully martensitic Ti-6Al-4V produced by ultra-fast cooling challenges the conventional strength–ductility trade-off and offers a new paradigm for designing structural alloys that combine high strength with high ductility.

## Figures and Tables

**Table 1:** Phase dimensions and fractions under different cooling rates.

Sample	Cooling Rate (°C/s)	Phase	Average Thickness (μm)	Average Length (μm)	Average Fraction (%)
Case 1	1	α	2.5 ± 1.0	325 ± 75	87.5 ± 2.5
		β	0.25 ± 0.15	325 ± 75	12.5 ± 2.5
Case 2	145	α_m_	8.0 ± 2.0	325 ± 75	35 ± 5
		Primary α′	2.5 ± 0.5	325 ± 75	17.5 ± 2.5
		Secondary α′	4.5 ± 0.5	7.5 ± 0.5	47.5 ± 2.5
Case 3	7000	Primary α′	2.5 ± 0.5	325 ± 75	7.5 ± 2.5
		Secondary α′	0.6 ± 0.2	4.5 ± 1.5	92.5 ± 2.5

**Table 2:** Compressive yield strain/stress and ultimate compressive strain/strength for different cases.

Specimens	Compositions (Phase)	Cooling Rate (°C/s)	ε_cy0.2_	ε_cu_	σ_cy0.2_ (MPa)	σ_cu_ (MPa)
Case 1	α/β	1	0.029	0.12	790.3	1160.5
Case 2	α_m_/α′	145	0.039	0.08	1214.7	1478.3
Case 3	α′	7000	0.037	0.17	1074.0	1519.2

**Table 3:** Calibrated elastic constants of β, α, and α′ phases for Ti-6Al-4V [21].

Elastic Constants (GPa)	*C*_11_	*C*_12_	*C*_13_	*C*_33_	*C*_44_
β	114	90	90	114	181
α	123	100	69	145	30
α′	120	100	67	125	30

**Table 4:** Calibrated Voce hardening parameters for different slip systems [21,38].

Hardening Parameters (MPa)	*τ*	*τ*_1_	*θ*_0_	*θ*_1_
β	41.09	99.62	500	20
α (basal)	253.5	5.0	700	350
α (prismatic)	354.8	5.0	700	350
α (pyramidal)	879.2	5.0	700	350
α′ (basal)	216.7	5.0	750	340
α′ (prismatic)	296.9	5.0	750	340
α′ (pyramidal)	822.3	5.0	750	340

## Data Availability

The original contributions presented in this study are included in the article. Further inquiries can be directed to the corresponding authors.
